# Association between neighborhood need and spatial access to food stores and fast food restaurants in neighborhoods of *Colonias*

**DOI:** 10.1186/1476-072X-8-9

**Published:** 2009-02-16

**Authors:** Joseph R Sharkey, Scott Horel, Daikwon Han, John C Huber

**Affiliations:** 1 Texas Healthy Aging Research Network (TxHAN) and Center for Community Health Development, School of Rural Public Health, Texas A&M Health Science Center, USA; 2 Program for Research in Nutrition and Health Disparities, School of Rural Public Health, Texas A&M Health Science Center, USA; 3 Department of Epidemiology and Biostatistics, School of Rural Public Health, Texas A&M Health Science Center, USA

## Abstract

**Objective:**

To determine the extent to which neighborhood needs (socioeconomic deprivation and vehicle availability) are associated with two criteria of food environment access: 1) distance to the nearest food store and fast food restaurant and 2) coverage (number) of food stores and fast food restaurants within a specified network distance of neighborhood areas of *colonias*, using ground-truthed methods.

**Methods:**

Data included locational points for 315 food stores and 204 fast food restaurants, and neighborhood characteristics from the 2000 U.S. Census for the 197 census block group (CBG) study area. Neighborhood deprivation and vehicle availability were calculated for each CBG. Minimum distance was determined by calculating network distance from the population-weighted center of each CBG to the nearest supercenter, supermarket, grocery, convenience store, dollar store, mass merchandiser, and fast food restaurant. Coverage was determined by calculating the number of each type of food store and fast food restaurant within a network distance of 1, 3, and 5 miles of each population-weighted CBG center. Neighborhood need and access were examined using Spearman ranked correlations, spatial autocorrelation, and multivariate regression models that adjusted for population density.

**Results:**

Overall, neighborhoods had best access to convenience stores, fast food restaurants, and dollar stores. After adjusting for population density, residents in neighborhoods with increased deprivation had to travel a significantly greater distance to the nearest supercenter or supermarket, grocery store, mass merchandiser, dollar store, and pharmacy for food items. The results were quite different for association of need with the number of stores within 1 mile. Deprivation was only associated with fast food restaurants; greater deprivation was associated with fewer fast food restaurants within 1 mile. CBG with greater lack of vehicle availability had slightly better access to more supercenters or supermarkets, grocery stores, or fast food restaurants. Increasing deprivation was associated with decreasing numbers of grocery stores, mass merchandisers, dollar stores, and fast food restaurants within 3 miles.

**Conclusion:**

It is important to understand not only the distance that people must travel to the nearest store to make a purchase, but also how many shopping opportunities they have in order to compare price, quality, and selection. Future research should examine how spatial access to the food environment influences the utilization of food stores and fast food restaurants, and the strategies used by low-income families to obtain food for the household.

## Background

**Figure 1:**
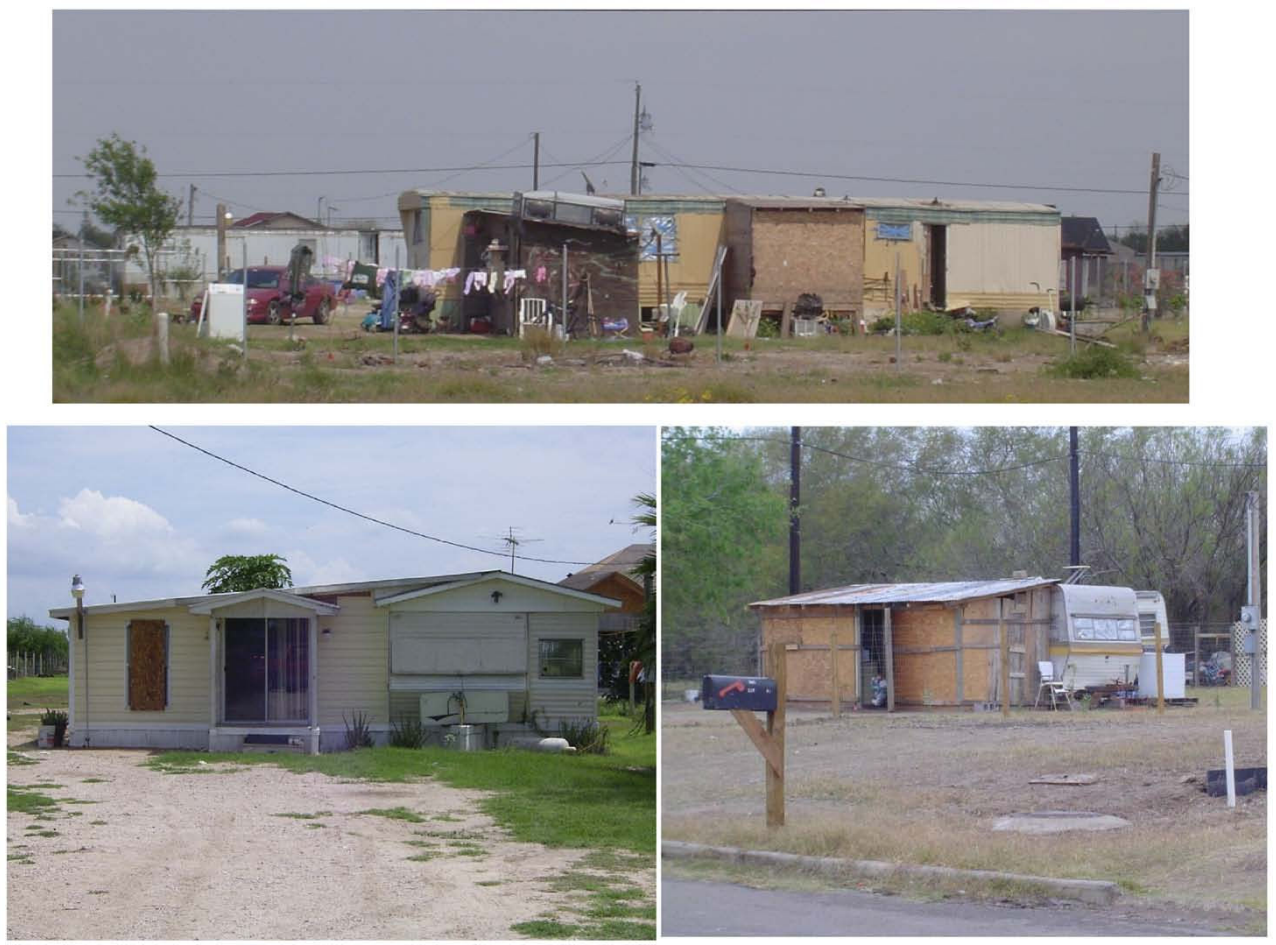
**Photographs taken in three *colonia *neighborhoods in the study area**.

The economic and social burdens posed by nutrition-related chronic health conditions (e.g., obesity, cardiovascular disease, and diabetes) are tremendous. It is well accepted that diet and nutrition influence health outcomes; much of the research focus has been on the relationship between nutrient intakes and disease processes [1]. The burden of nutrition-related conditions becomes greater for marginalized populations that face greater vulnerability to food insecurity, poor nutritional health, and adverse health outcomes [2]. One, such marginalized population is Hispanic families who reside in *colonias *along the Texas-Mexico border and are primarily poor. *Colonias*, which were developed from subdivided agricultural lands in response to a deficit in low-income housing [3], are substandard residential areas with inadequate roads, variable housing conditions (see Figure 1), and drainage which frequently does not provide access to safe water or sewer sources [4]. Almost 20% of these largely Hispanic households have a female household head, and half of all children are food insecure [5]. Almost 70% of all *colonias *in the Lower Rio Grande Valley of Texas are located in Hidalgo County, where the number of *colonias *continues to grow [6]. Residents of *colonias *face great structural and neighborhood disadvantage, such as inadequate roads, limited or non-existent public transportation, and poor access to community resources [7]. This makes it particularly difficult for children and adults in these areas to initiate or maintain healthy eating habits that are critical for the prevention of disease conditions.

Ecological approaches to behavior change and health recognize that there is a dynamic interaction between the individual and the environment [2,8,9]. For example, the consumption of healthy foods, such as fruits and vegetables, are recommended for overall nutritional health; however, they are often not easily accessible [10]. Local food environments, which are a primary source for food that is consumed, may have an effect upon health and well-being through food cost and availability [11-14]. In documenting a relationship between food access and health, studies have used several proxy measures for health: 1) access to fruits and vegetables or other healthy foods, 2) intake of fruits and vegetables, and 3) prevalence of obesity. In a Colorado study that involved data collection at multiple times, greater availability of produce was associated with greater increase in fruit and vegetable servings [15]. In a review of 12 descriptive studies, Robinson found that dietary behaviors and fruit and vegetable intake among African Americans in the U.S. were the result of several factors, including food access and availability [9]. Using data from the Atherosclerosis Risk in Communities (ARIC) study, Morland and colleagues found that regardless of race, fruit and vegetable intake was higher in census tracts with more supermarkets [11]. Additional observational studies supported this relationship [16-22]. In a study of urban New Orleans, Bodor and colleagues found that greater availability of fresh vegetables in the neighborhood, regardless of type of store, was associated with increased intake [23]. In contrast, a postal survey conducted among 426 respondents to a postal survey (42.6% response rate) in the U.K. found that travel distance to the nearest supermarket was not associated to either fruit or vegetable consumption [24]. In two U.K. natural experiments that involved the introduction of a new supermarket in an area that previously had limited access to food stores, the results were not consistent on a relationship between food access and consumption [25,26]. Morland and colleagues looked at the relationship between food stores and obesity, using ARIC data, and found that obesity was associated with lower numbers of supermarkets and higher numbers of convenience stores within census tracts [27]. A large study in urban areas of Massachusetts found a significant association between environmental variables, such as presence of a supermarket, and obesity [28]. When considering away-from-home foods, such as fast food, Thomson and colleagues found a relationship between frequency of fast food consumption and development of obesity among U.S. girls [29].

However, the results of food environment studies from the U.S., U.K., Canada, Australia, and New Zealand that examined physical access to food stores are mixed. Some studies determined that families who live in poorer areas (e.g., lower socioeconomic or greater deprivation) or minority neighborhoods have little or no access to supermarkets, where there are larger selections of healthy foods (e.g., fruits, vegetables, low-fat dairy and meats) [12,30-39]. Other studies found little or no difference in access to supermarkets between deprived and affluent areas [32,40-45], or better access to food stores from more deprived neighborhoods [46-49]. Although the availability of healthy foods in food stores or consumption of healthy foods by residents is beyond the scope of this paper, it stands to reason that proximity of local food environments may influence food choice and adherence to dietary recommendations through food cost and availability [11-14,18,50].

Individual and community concerns with food security, limited access to supermarkets, higher costs for food (food price and transportation cost), and nutritional status are receiving increased attention [12,18,51-53]. However, there has been limited study related to: 1) use of multiple dimensions of spatial access to food stores, such as proximity (minimum distance) and coverage (number within a specified area), especially in the U.S. [14,22,35,38,40,46,49,54]; 2) spatial equity – the distribution of food resources in relation to population need [46,55-59]; 3) the growing influence of convenience stores and non-traditional food stores, such as mass merchandisers (e.g., Target, Kmart, Wal-Mart) and dollar stores on food availability [45,60]; and 4) the increased reliance on fast-food meals for in-home and away-from-home consumption [42,61,62].

Using network-based spatial measures, our primary objective was to determine the extent to which neighborhood need is associated with spatial access to food stores and fast food outlets in areas of *colonias *by 1) identifying and geocoding all food stores and fast food outlets in a 197-census block group (CBG) area of Hidalgo County, using ground-truthed methods (direct observation and on-site GPS); 2) determining network-based potential spatial access using distance and coverage criteria; and 3) using multivariate models to examine the relationship between neighborhood need and potential spatial access to food stores and fast food outlets.

## Methods

### Study Area

**Figure 2:**
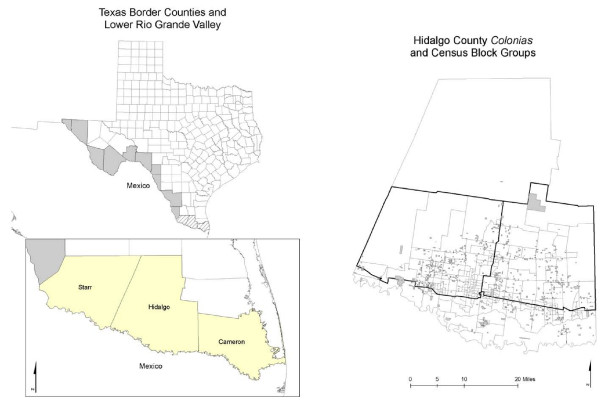
**Map of Texas and Hidalgo County study area with *colonias *neighborhoods**. Study area indicated by darkened border.

The study used data from the 2006–2007 *Colonias *Food Environment Project (CFEP), which was approved by the Institutional Review Board at Texas A&M University, and the decennial 2000 U.S. Census Summary File 3 (SF-3). The CFEP is a comprehensive study of the food environment that used ground-truth methods in 197 CBGs of Hidalgo County in the Lower Rio Grande Valley of Texas (see Figure 2). The land area for the study encompasses 772 mi^2^, and includes approximately 80% of all *colonias *in the county. According to the 2000 U.S. Census, 88% of the population in the study area self-identified as Hispanic/Latino; and more than 83% speak a language other than English at home [63].

### Direct Measurement of Food Environment

Using a modified version of the 2002 North America Industry Classification System (NAICS) definitions [46,64], three overall categories of food stores were identified: traditional food stores included supercenters, supermarkets, and grocery stores; convenience food stores included convenience stores and food marts; and non-traditional food stores were mass merchandisers (e.g., Kmart, Target, and Wal-Mart), dollar stores, and chain drug stores or pharmacies [65]. The following definitions were used to classify specific types of food stores by outside observation. Supercenters or superstores are very large stores that primarily engage in retailing a general line of groceries in combination with general lines of new merchandise, such as apparel, furniture, and appliances (e.g., Super Wal-Mart, Super Kmart). Although supermarkets and grocery stores primarily engage in retailing a general line of food, supermarkets are larger in size (>20,000 ft^2^), number of employees, and sales volume [30]. In this study, chain store identification and number of parking spaces (>100) were used to distinguish supermarkets from grocery stores [65]. Convenience stores or food marts primarily engage in retailing a limited line of goods that generally includes milk, bread, soda, and snacks. The convenience store category also included convenience stores with gasoline and gasoline stations with convenience stores. Mass merchandisers were large, general merchandise "value" stores, such as Kmart, Target, and Wal-Mart. Dollar stores are limited price general merchandise "value" stores, such as Dollar General or Family Dollar [66]. Pharmacies and drug stores that were part of national chains were included (e.g., CVS, Walgreens). Fast food restaurants were defined as limited-service restaurants where patrons order or select items and pay before eating. Since the fast food category of restaurants is more commonly associated with marketing less than healthy food items, other restaurant categories, such as full-service restaurants, which are seldom utilized by *colonia *residents [67], were excluded from study [68-70]. Using ground-truthed methods developed in a prior study and shown to be more accurate than the use of readily available data sources, food stores and fast food restaurants were identified by systematically driving all U.S., State, Farm-to-Market, and city/town roads in the study area [46]. Global Positioning System data were collected in front of each location using a Bluetooth Wide Area Augmentation System (WAAS)-enabled GPS receiver and the latest World Geodetic System 1984 (WGS 84) datum. Locational points were determined after at least four satellite signals were detected. WAAS-enabled GPS has been shown to be significantly more accurate than typical, non-WAAS GPS [71], with positional accuracy of <3 meters [72].

### Potential Spatial Access

Neighborhoods were characterized by CBG, which represent the smallest geographic unit of the census from which detailed "long form" social and economic data are tabulated [73,74]. Two criteria of spatial access were calculated from the population-weighted centroid (population center) of each CBG [46,75]: 1) proximity, and 2) coverage [40]. Proximity: ESRI's Network Analyst extension in ArcInfo 9.2 was used to calculate the shortest network distance along the road network between paired point data (population-weighted CBG centroid and the nearest corresponding food store or fast food restaurant within the study area). Separate distances were calculated from each CBG to each type of shopping opportunity – the nearest supercenter, supermarket, grocery store, convenience store, mass merchandiser, dollar store, pharmacy, and fast food restaurant in miles. Since both supercenters and supermarkets offer greater selections of foods, especially healthy options, and lower prices than other food store formats [14,76], an additional distance measure was calculated to nearest supercenter or supermarket, which was defined as supercenters/supermarkets [35]. Coverage: Network Analyst computed the total number of each type of food store and fast food restaurant within a one-mile, three-mile, and five-mile buffer, using the shortest network distance from the population-weighted center of each CBG. Since the study area is not a large, highly dense urban area as much of the limited literature describes (e.g., Chicago, Detroit, Montreal, Los Angeles) [35,40,50], coverage distances were selected that represented a long walk (1 mile) and reachable by car (within 3 and 5 miles). Proximity measured the shortest distance needed to travel to a specific type of food store or fast food restaurant, while coverage indicates the number of shopping opportunities. More opportunities equates to greater accessibility [77].

### Neighborhood Need

Using the 2000 SF-3, two socioeconomic indicators were used to estimate neighborhood need for access to food stores and fast food restaurants: 1) neighborhood socioeconomic deprivation, and 2) the percentage of occupied households without an available vehicle. Neighborhoods with greater socioeconomic deprivation or lower vehicle availability are likely to have a greater need for access to food stores. An additional variable – population density (total population per square mile) – was calculated to characterize neighborhoods [35].

#### Neighborhood socioeconomic deprivation

Seven CBG socioeconomic measures were extracted from the SF-3 that represented neighborhood unemployment, telephone service, public assistance, complete kitchen, complete plumbing, low education attainment, and poverty. Using established procedures, CBG data were merged and a factor analysis, using iterated principal factor method (Release 9, 2005, Stata Statistical Software), was constructed to reduce the number of linear combinations and to identify an overall index of neighborhood socioeconomic deprivation [46,58,78-80]. One factor (eigenvalue 3.7) was identified and provided item loadings (in parenthesis), which were used to weight each variable's contribution to the deprivation summary score [58,78,79]: percent unemployed (0.52), percent of households without telephone service (0.60), percent of families receiving public assistance (0.70), percent of households lacking complete kitchen facilities (0.79), percent of households lacking complete plumbing facilities (0.79), percent 25-years or older and with less than 10 years education (0.83), and percent living below the poverty threshold (0.81). The internal consistency of this measure was good (Cronbach's α = 0.79). The neighborhood socioeconomic deprivation (deprivation) index was standardized by dividing the index by the square of the eigenvalue [58,81].

### Statistical Analysis

Release 9 of Stata Statistical Software was used for all statistical analyses; *p *< 0.05 was considered statistically significant. Descriptive statistics were estimated for the accessibility and need indicators. Spearman rank correlation method was used to test the direction and strength between access criteria and need indicators. Because nearby neighborhoods are more likely to have similar access characteristics than distant ones, we examined spatial nature of the data using spatial autocorrelation measures – i.e. how neighborhoods are spatially correlated in terms of access to different food stores. Moran's I statistics, one of the most commonly used measures of global spatial autocorrelation, were obtained for both distance and coverage methods of access measures. Spatial weight matrix was constructed based on binary connectivity (adjacency), and expected I, variance, and z-scores were obtained to assess significance of the correlation in Geoda [82]. Moran's I values range between -1 and 1, with values near 1 indicating highly positive spatial autocorrelation of similar access characteristics.

Finally, a single multivariate regression model was fitted to determine the relationship of neighborhood need (deprivation and vehicle availability) to potential spatial access to food stores and fast food restaurants, controlling for population density. The multivariate model was chosen instead of seven separate multiple regression models (one for each outcome variable) for two reasons: 1) the seven outcome variables are correlated with each other and the multivariate regression accounts for this correlation when testing hypotheses about the predictor variables; and 2) the final collection of models is easier to interpret if the same predictor variables are identified for all seven types of food stores and fast food restaurants.

## Results

**Figure 3:**
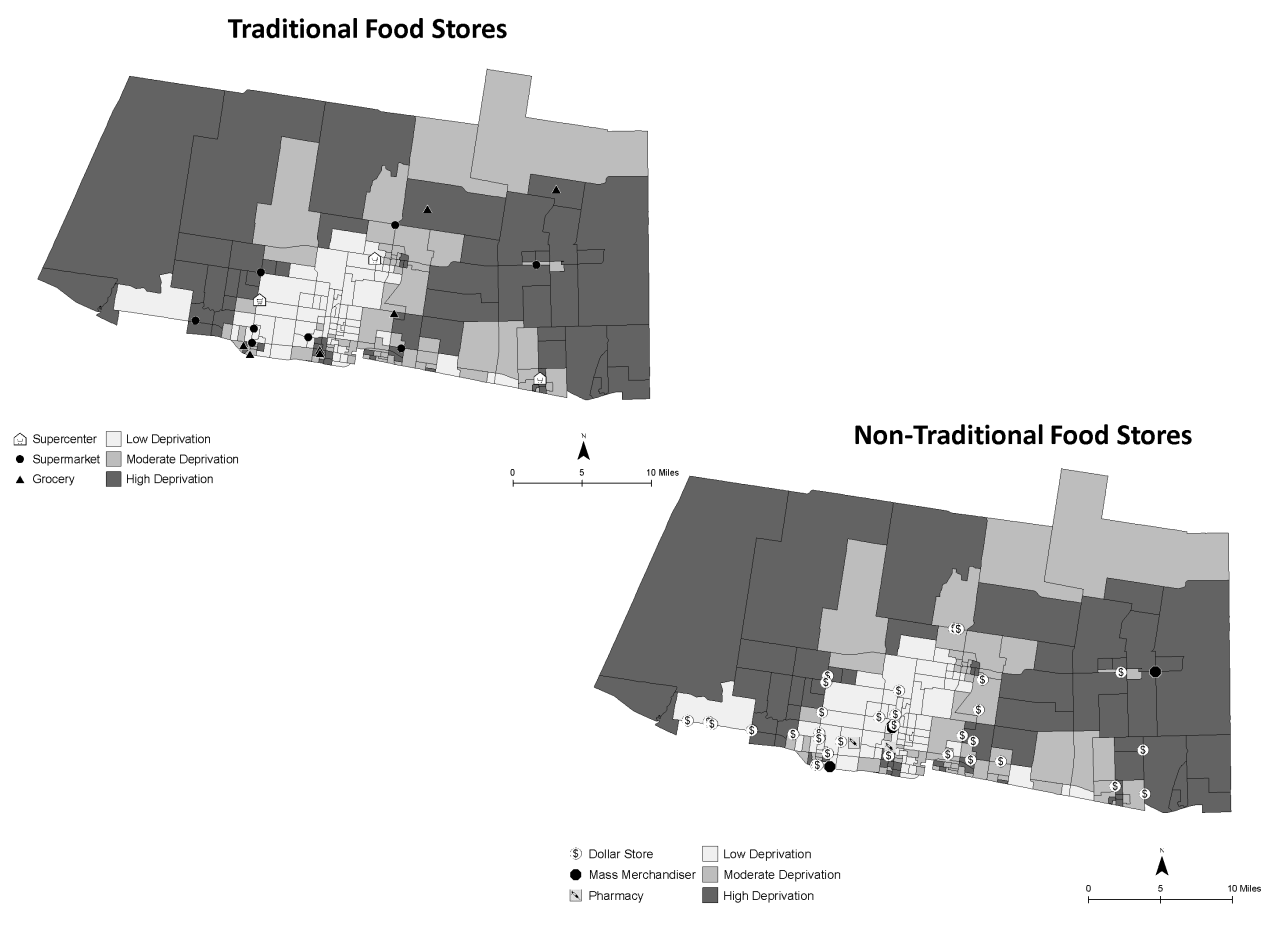
**Spatial distribution of traditional and non-traditional food stores using on-site GPS data**. Census block groups are shaded to indicate the level of deprivation.

**Figure 4:**
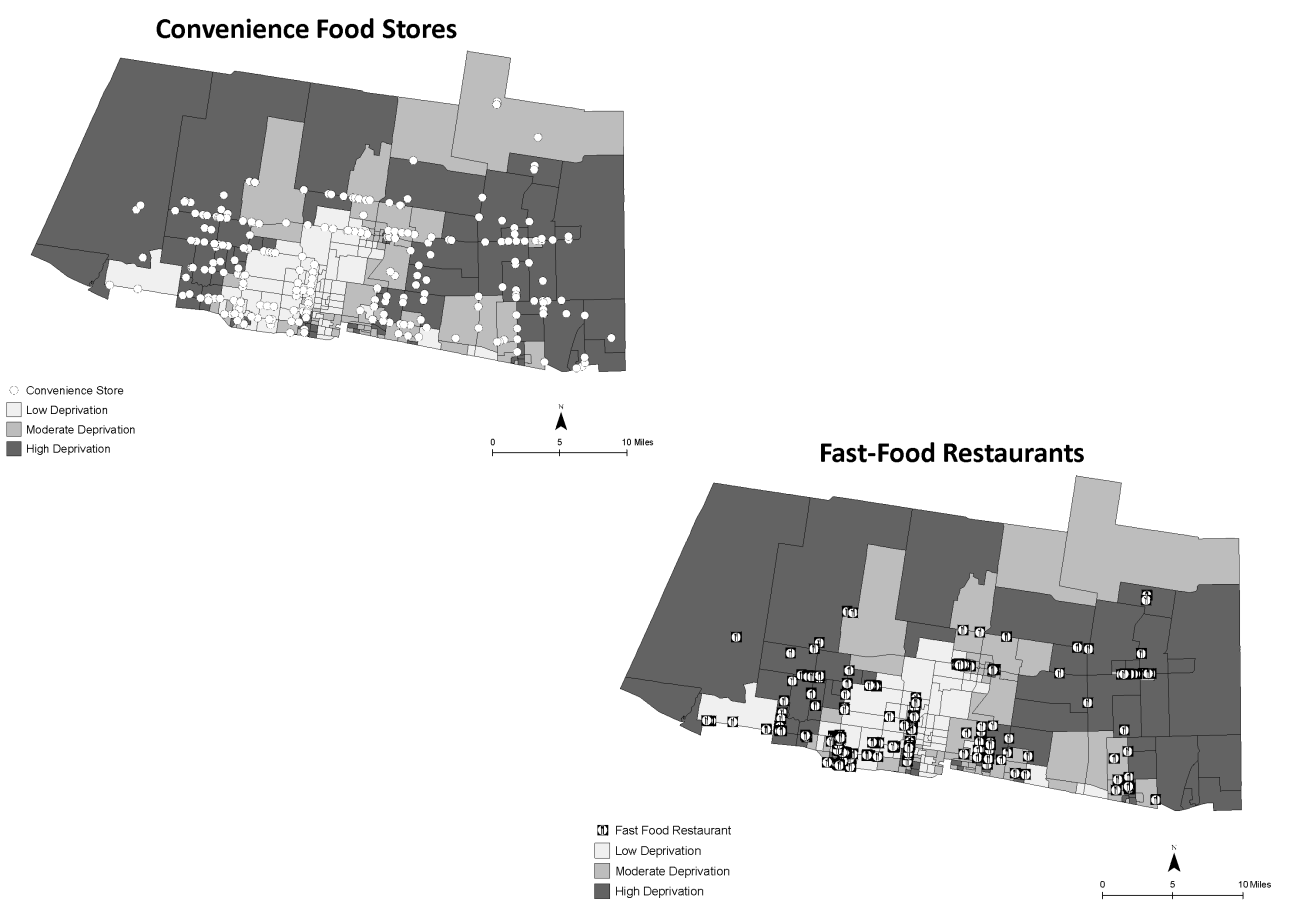
**Spatial distribution of convenience food stores and fast food restaurants using on-site GPS data**. Census block groups are shaded to indicate the level of deprivation.

There were 315 food stores (21 traditional food stores, 255 convenience stores, and 39 non-traditional food stores) and 204 fast food restaurants identified and geocoded. Traditional food stores included 3 supercenters, 11 supermarkets, and 7 grocery stores, while non-traditional food stores included 33 dollar stores, 4 mass-merchandisers, and 2 pharmacies. Forty-four percent of the fast food restaurants were national chains. Figures 3 and 4 show the spatial distribution of food stores, fast food restaurants, and CBG deprivation. Figure 3 shows that few food stores are located in high deprivation areas. In contrast, Figure 4 shows that convenience food stores and fast food restaurants can be found in areas of high deprivation.

The distribution of neighborhood socioeconomic characteristics shows diverse levels of neighborhood need in the study area (Table 1). In data not shown, in 25% of the 197 CBG, at least 16% of households did not have an available vehicle; and in 10% of CBG, at least 24% of households were without an available vehicle. Table 1 also shows the distribution of proximity and coverage. Considering both proximity and coverage, neighborhoods had best access to convenience stores, fast food restaurants, and dollar stores.

**Table 1:** Neighborhood need indicators and spatial accessibility to food stores and fast food restaurants*

	Mean ± SD	Median	Minimum	Maximum
**Neighborhood Need**				
***Neighborhood socioeconomic characteristics, %***				
Residents in poverty^†^	33.9 ± 16.1	34.0	0	69.0
Public assistance^†^	10.8 ± 7.1	10.2	0	38.6
Unemployment^†^	6.5 ± 3.5	6.1	0	18.2
Households with no telephone service^†^	2.8 ± 3.1	2.1	0	13.3
Households that lack complete plumbing facilities^†^	3.3 ± 4.4	1.5	0	27.5
Households that lack complete kitchen facilities^†^	2.9 ± 3.7	1.4	0	16.8
<10^th ^grade education^†^	39.1 ± 18.3	42.5	2.8	74.0
Households without available vehicle	11.5 ± 9.5	8.4	0	53.0
Population density	3189.0 ± 2473.7	2528.9	22.1	11511.1
**Spatial Accessibility**				
***Proximity (minimum distance in miles)***^**‡**^				
Traditional Food Stores				
Supercenter	5.93 ± 3.17	5.92	0.57	19.34
Supermarket	3.71 ± 2.70	3.02	0.15	14.06
Supercenter/Supermarket	2.84 ± 1.85	2.56	0.15	11.26
Grocery Store	5.11 ± 4.05	3.99	0.19	18.53
Convenience	1.03 ± 0.81	0.85	0.02	5.63
Non-Traditional Food Stores				
Dollar Stores	2.03 ± 1.52	1.60	0.16	11.69
Mass Merchandisers	6.08 ± 3.82	6.01	0.28	17.58
Pharmacy	8.25 ± 6.50	6.43	0.50	27.70
Fast Food Restaurants	1.35 ± 1.19	0.97	0.09	7.51
				
***Coverage – 1 Mile***^**§**^				
Traditional Food Stores				
Supercenter	0.07 ± 0.25	0.00	0.00	1.00
Supermarket	0.17 ± 0.43	0.00	0.00	2.00
Supercenter/Supermarket	0.27 ± 0.55	0.00	0.00	2.00
Grocery Store	0.22 ± 0.60	0.00	0.00	2.00
Convenience Food Stores	2.99 ± 2.65	2.00	0.00	10.00
Non-Traditional Food Stores				
Dollar Stores	0.56 ± 0.78	0.00	0.00	3.00
Mass Merchandisers	0.10 ± 0.40	0.00	0.00	2.00
Pharmacy	0.07 ± 0.25	0.00	0.00	1.00
Fast Food Restaurants	4.33 ± 5.46	2.00	0.00	24.00
				
***Coverage – 3 Miles***^**¶**^				
Traditional Food Stores				
Supercenter	0.28 ± 0.45	0.00	0.00	1.00
Supermarket	0.81 ± 0.72	1.00	0.00	3.00
Supercenter/Supermarket	1.19 ± 1.05	1.00	0.00	5.00
Grocery Store	0.73 ± 0.91	0.00	0.00	3.00
Convenience Food Stores	18.29 ± 7.98	18.00	0.00	39.00
Non-Traditional Food Stores				
Dollar Stores	3.15 ± 2.15	3.00	0.00	9.00
Mass Merchandisers	0.60 ± 0.85	0.00	0.00	3.00
Pharmacy	0.41 ± 0.64	0.00	0.00	2.00
Fast Food Restaurants	20.73 ± 12.96	20.00	0.00	60.00
				
***Coverage – 5 Miles***^**#**^				
Traditional Food Stores				
Supercenter	0.56 ± 0.52	1.00	0.00	2.00
Supermarket	1.58 ± 1.27	1.00	0.00	5.00
Supercenter/Supermarket	2.31 ± 1.79	2.00	0.00	7.00
Grocery Store	1.64 ± 1.27	2.00	0.00	4.00
Convenience Food Stores	42.25 ± 15.40	43.00	0.00	82.00
Non-Traditional Food Stores				
Dollar Stores	7.05 ± 3.61	7.00	0.00	15.00
Mass Merchandisers	1.06 ± 1.13	1.00	0.00	3.00
Pharmacy	0.81 ± 0.90	0.00	0.00	2.00
Fast Food Restaurants	42.70 ± 23.53	43.00	0.00	96.00

* Values calculated for each of the CBG (census block group) in the study area (*n *= 197).

^† ^Items included in the Neighborhood Socioeconomic Deprivation index

^‡ ^Network distance calculated from the population-weighted centroid of each CBG

^§ ^Number of food stores or restaurants within 1 network mile of the CBG population-weighted centroid

^¶ ^Number of food stores or restaurants within 3 network miles of the CBG population-weighted centroid

^# ^Number of food stores or restaurants within 5 network miles of the CBG population-weighted centroid

### Spatial Accessibility and Need

Table 2 shows the correlation between potential spatial accessibility and need. Positive values of proximity coefficients denote greater minimum distance and poorer accessibility; and positive values of coverage coefficients denote greater diversity of shopping opportunities [40]. According to proximity criterion, neighborhoods of greater deprivation had significantly better access to a convenience store and poorer access to food from supercenters, grocery stores, mass merchandisers, and pharmacies. Neighborhoods with greater proportion of households without an available vehicle had better access to food from a supercenter/supermarket, convenience store, dollar store, and fast food restaurant. Considering both criteria of access, the results were inconsistent for access to grocery stores within one network mile; for more deprived neighborhoods, the distance to the nearest grocery store was further; access to multiple grocery stores within one mile was better. However, as the distance from the neighborhood increased, the relationship between deprivation and grocery stores became consistent in direction with the distance measure. Within one network mile, neighborhoods with a greater proportion of households without a vehicle had a greater number of opportunities for food from supermarkets, supercenter/supermarkets, grocery stores, convenience stores, and fast food restaurants. This became relatively fewer opportunities within 5 miles.

**Table 2:** Spearman ranked correlations between potential spatial accessibility and neighborhood need

	**Proximity**		***Coverage***					
			***One Mile***		***Three Mile***		***Five Miles***	
	Deprivation	Vehicle	Deprivation	Vehicle	Deprivation	Vehicle	Deprivation	Vehicle

Traditional FS								
Supercenter	0.169*	0.105	0.019	0.125	-0.054	0.052	**-0.173**^†^	**-0.215**^†^
Supermarket	0.044	-0.134	0.044	0.144*	-0.028	0.108	**-0.223**^‡^	**-0.171**^†^
Supercenter/Supermarket	0.046	**-0.223**^‡^	0.053	**0.214**^‡^	-0.043	0.142	**-0.278**^‡^	**-0.228**^‡^
Grocery	**0.253**^‡^	-0.080	**0.208**^‡^	**0.347**^‡^	**-0.289**^‡^	-0.023	**-0.442**^‡^	-0.058
Convenience	**-0.245**^‡^	**-0.271**^‡^	0.110	**0.177**^†^	-0.099	-0.056	**-0.297**^‡^	**-0.297**^‡^
Non-Traditional FS								
Dollar Store	0.105	**-0.178**^†^	-0.052	0.073	**-0.373**^‡^	-0.137	**-0.409**^‡^	**-0.263**^‡^
Mass Merchandiser	**0.398**^‡^	0.166*	**-0.201**^†^	-0.082	**-0.444**^‡^	-0.126	**-0.406**^‡^	-0.171*
Pharmacy	**0.472**^‡^	**0.159**^†^	-0.087	-0.001	**-0.404**^‡^	-0.097	**-0.468**^‡^	-0.171*
Fast Food Restaurants	-0.044	**-0.237**^‡^	-0.083	**0.203**^†^	**-0.364**^‡^	-0.064	**-0.404**^‡^	**-0.255**^‡^

NOTE: Higher values (positive numbers) of proximity denote poorer accessibility; higher values (positive numbers) of coverage denote greater accessibility.

Bold values indicate statistical significance.

Deprivation, Neighborhood Socioeconomic Deprivation; Vehicle, % households without an available vehicle; FS, Food Store

*p < 0.05 for correlation between accessibility and need

^†^*p *< 0.01 correlation between accessibility and need

^‡^*p *< 0.001 correlation between accessibility and need

**Figure 5:**
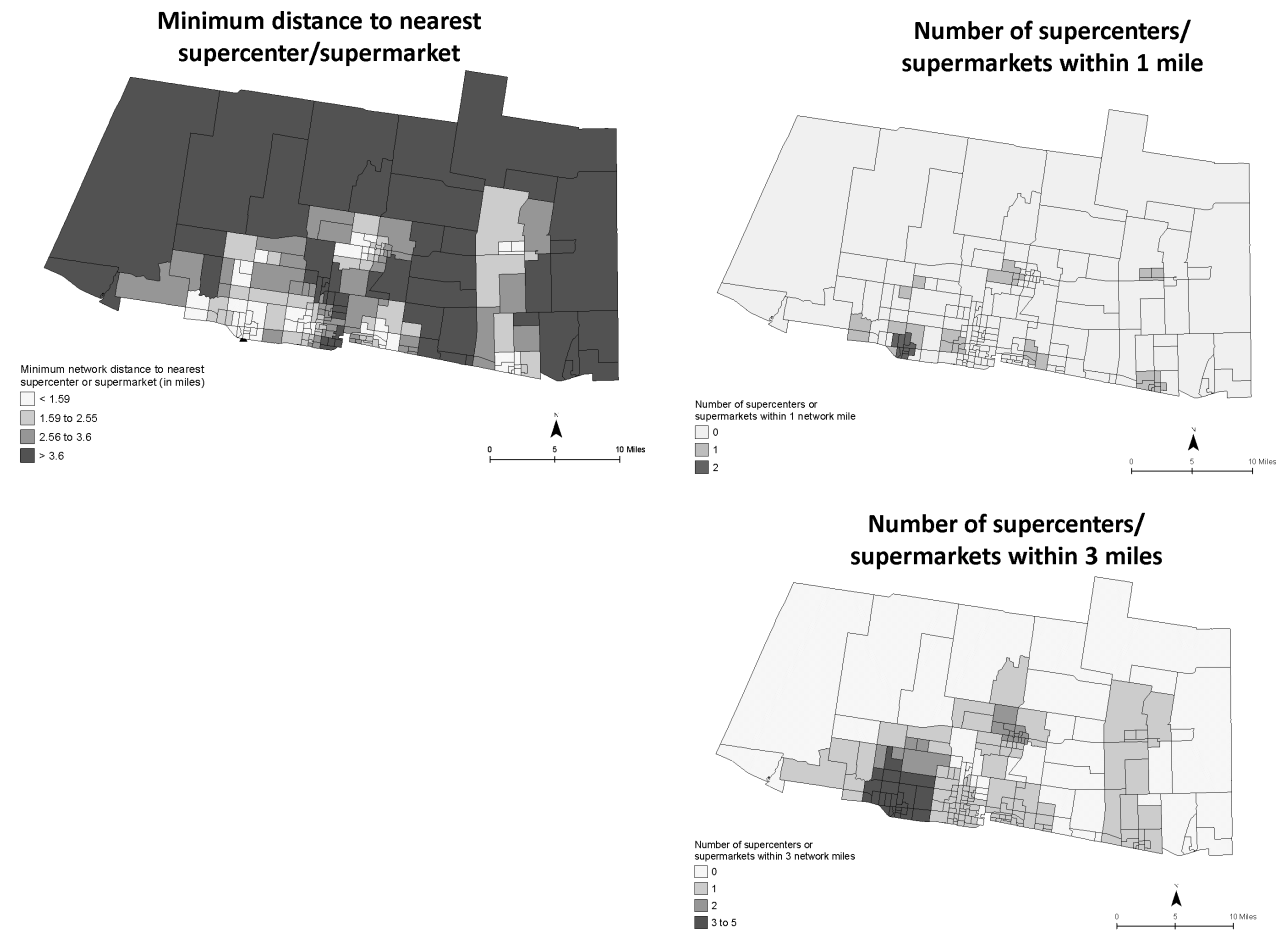
**Neighborhood accessibility of supercenter/supermarket using minimum distance to the nearest location and variety (number of locations within 1- and 3-mile network distance)**. CBG are shaded to indicate relative distance (darker = greater distance) and variety (darker = greater variety).

**Figure 6:**
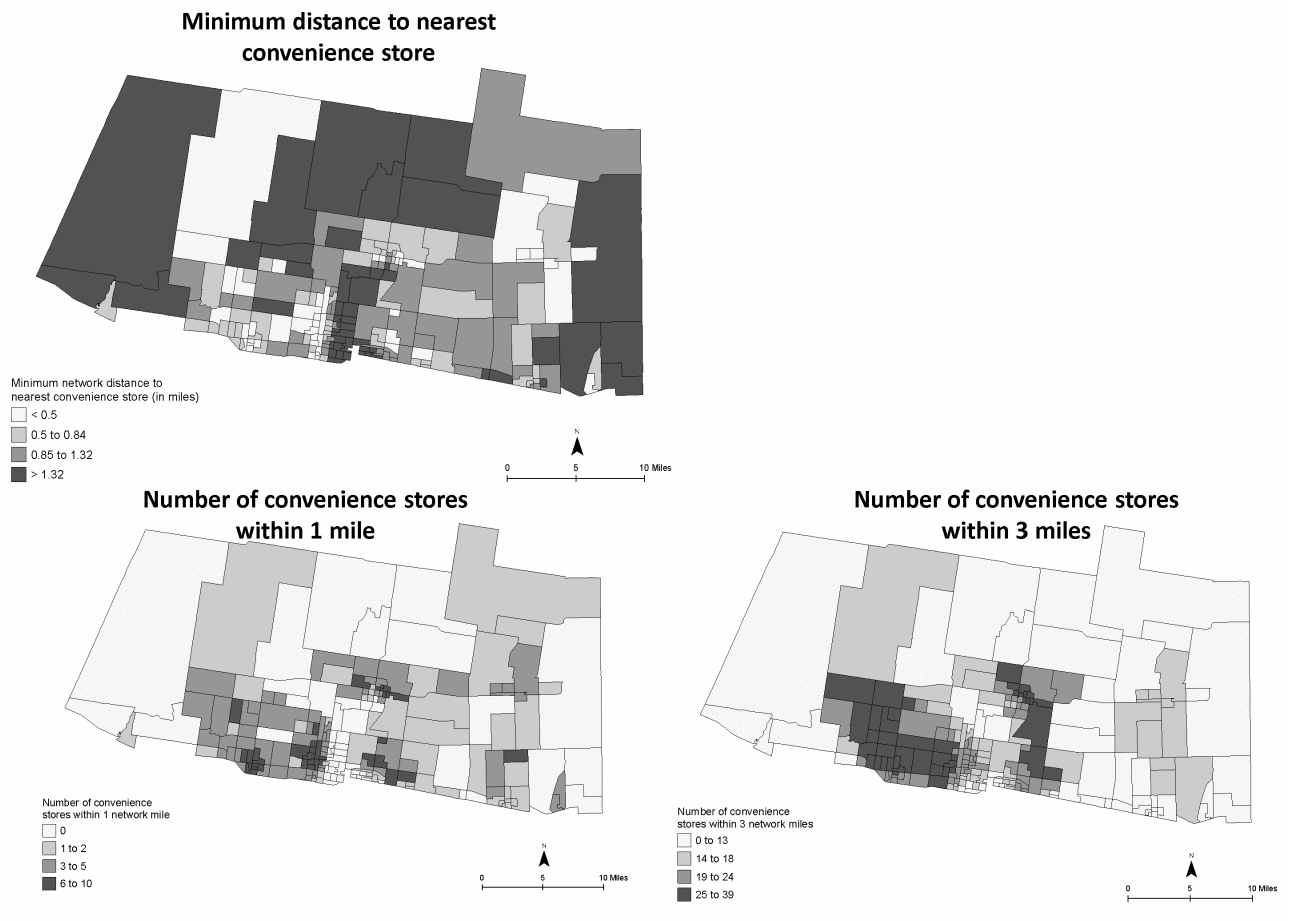
**Neighborhood accessibility of convenience stores using minimum distance to the nearest location and variety (number of locations within 1- and 3-mile network distance)**. CBG are shaded to indicate relative distance (darker = greater distance) and variety (darker = greater variety).

**Figure 7:**
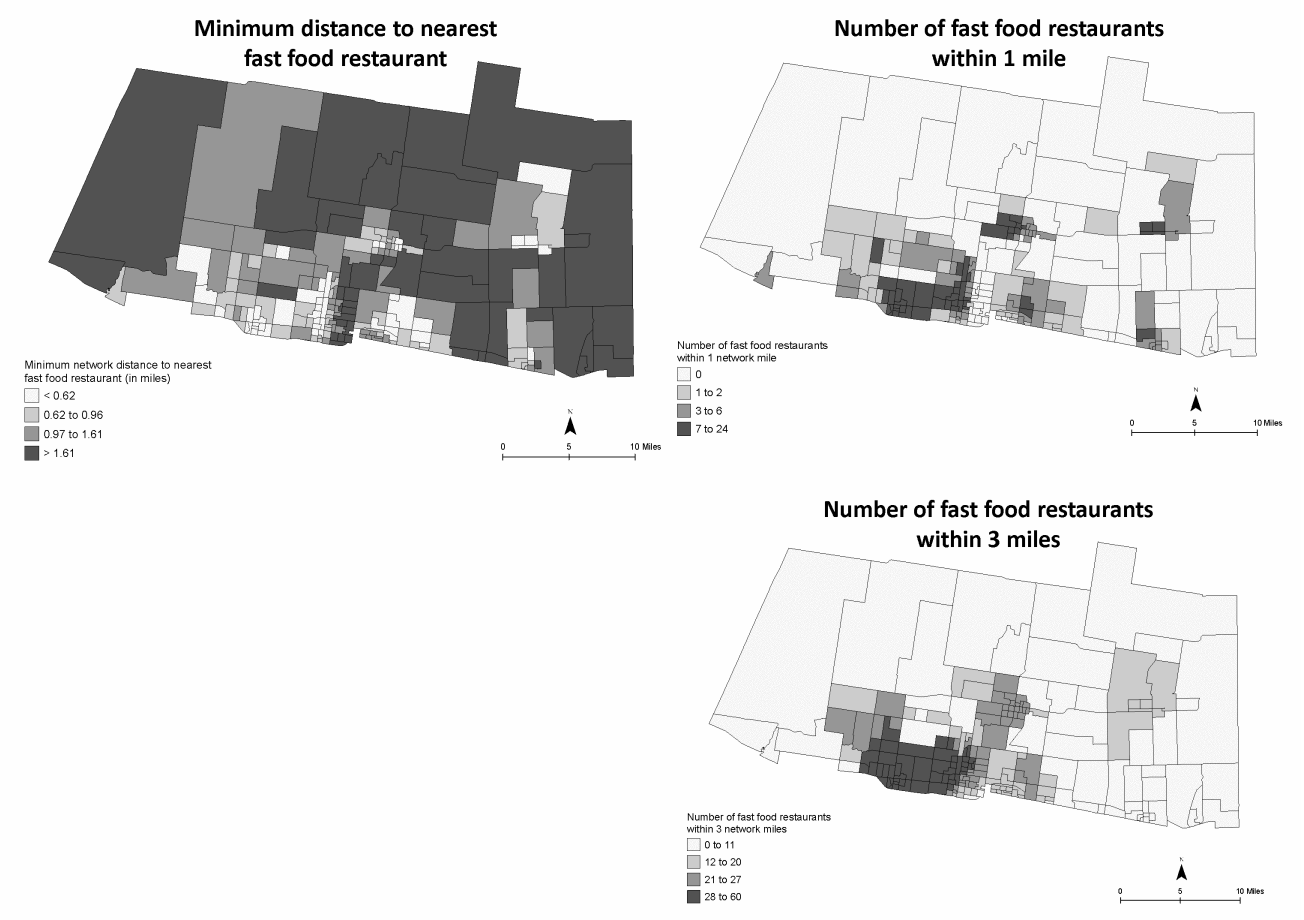
**Neighborhood accessibility of fast food restaurants using minimum distance to the nearest location and variety (number of locations within 1- and 3-mile network distance)**. CBG are shaded to indicate relative distance (darker = greater distance) and variety (darker = greater variety).

Accessibility to supercenters/supermarkets, convenience stores, and fast food restaurants were mapped using both proximity and coverage measures (Figures 5, 6, 7). Figure 5 shows similar inaccessibility to a supercenter or supermarket, using both measures. The nearest supercenter or supermarket is more than 3.6 miles (one way) from 25% of the CBG; more than 78% of CBG (*n *= 154) do not have access to one supercenter or supermarket within one mile; and 21.8% (*n *= 43) of CBG remain without access within three miles. In data not shown, 70.6% of CBG (*n *= 139) do not have a supercenter, supermarket, or small grocery within one mile; 29% of these CBG are considered high deprivation. Figures 6 and 7 depict both measures for access to convenience stores and fast food restaurants. Overall, neighborhoods had better access to convenience stores and fast food restaurants. However, more than 25% (*n *= 50) CBG do not have a convenience store within one mile; 49 of these CBG were also void of a supercenter, supermarket, or grocery store within one mile (data not shown). On the other hand, 75% of the study area had at least 14 convenience stores and 12 fast food restaurants within three miles. In data not shown, neighborhoods with lower population density had greater distance to nearest food store (all types) and fast food restaurant, and had less coverage of food stores within 1-mile and 3-mile buffers.

Moran's I and z-scores for minimum distance, and 1-, 3-, 5-mile coverage methods are presented in Table 3. Overall we found evidence of spatial autocorrelation among nearby neighborhoods. Moran's I values are relatively higher for supercenters, supermarkets, grocery stores, mass merchandisers, and pharmacies, and lower for supercenter/supermarket, convenience store, dollar store, and fast food restaurants. This implies that, at least for the measure of proximity for supercenter/supermarket, convenience store, dollar store, and fast food restaurants, areas with similar values are less likely to be clustered in space. Based on the Moran's I values for the number of stores within 1 mile, the inference of less clustering in space for all stores could be made. However, we should be cautious interpreting this because it may be due to the fact that there are only small numbers of food stores within 1 mile.

**Table 3:** Spatial autocorrelation statistics for accessibility measures (distance and coverage)

	Distance to the nearest (in miles)		Number of stores within 1 network mile		Number of stores within 3 network miles		Number of stores within 5 network miles	
	**Moran's I**	**Z-score**	**Moran's I**	**Z-score**	**Moran's I**	**Z-score**	**Moran's I**	**Z-score**
Supercenter	0.47	11.52	0.18	3.89	0.40	8.56	0.48	10.96
Supermarket	0.50	10.41	0.25	5.66	0.52	12.06	0.62	13.53
Supercenter/supermarket	0.24	5.46	0.20	4.53	0.50	11.08	0.62	14.17
Grocery store	0.61	13.57	0.19	4.49	0.67	14.60	0.71	15.69
Convenience store	0.23	5.37	0.29	6.32	0.42	9.34	0.41	9.73
Mass merchandiser	0.70	15.17	0.17	3.95	0.69	15.57	0.66	14.97
Dollar store	0.32	7.19	0.39	8.28	0.58	12.59	0.62	13.69
Pharmacy	0.73	15.74	0.21	4.86	0.71	15.35	0.83	17.51
Fast food restaurant	0.20	4.51	0.30	6.94	0.59	12.90	0.66	14.59

#### Multivariate Models for Access

Multivariate linear regression models were used to examine the relationship between area deprivation, vehicle availability and access to food stores and fast food restaurants, controlling for population density. Table 4 shows that, adjusting for population density, residents in neighborhoods with increased deprivation had to travel a significantly greater distance to the nearest supercenter/supermarket, grocery store, mass merchandiser, dollar store, and pharmacy. The results were quite different for association of need with the number of stores within 1 mile (Table 5). Deprivation was only associated with fast food restaurants; greater deprivation was associated with a fewer number of fast food restaurants within 1 mile. CBG with greater lack of vehicle availability had slightly better access to more supercenters/supermarkets, grocery stores, or fast food restaurants. Vehicle availability was not associated with variety of food venues within 3 miles (Table 6); however, increasing deprivation was associated with decreasing numbers of grocery stores, mass merchandisers, dollar stores, and fast food restaurants within 3 miles.

**Table 4:** Association between proximity and need, using multivariate linear regression

	Access as network distance to the nearest						
	
	Supercenter/Supermarket	Grocery Store	Convenience Store	Mass Merchandiser	Dollar Store	Pharmacy	Fast Food Restaurant
	
Need variable	b (SE)	b (SE)	b (SE)	b (SE)	b (SE)	b (SE)	b (SE)
Deprivation	0.133 (0.053)^†^	0.475 (0.115)^‡^	-0.031 (0.025)	0.537 (0.105)^‡^	0.121 (0.041)^†^	1.061 (0.161)^‡^	0.053 (0.034)
No Vehicle	-0.029 (0.016)	-0.024 (0.035)	-0.005 (0.007)	0.012 (0.032)	-0.015 (0.012)	0.032 (0.049)	-0.009 (0.010)

R^2^	0.204	0.213	0.085	0.252	0.274	0.397	0.206
*P*	<0.001	<0.001	<0.001	<0.001	<0.001	<0.001	<0.001

NOTE: In this model, the seven equations were simultaneously estimated, controlling for population density. All variable entered as continuous. Results are reported as multivariate-adjusted b (SE). Statistically significant variables are indicated as: *<0.05 ^†^<0.01 ^‡^<0.001

**Table 5:** Association between 1-mile coverage and need, using multivariate linear regression

	Access as coverage within 1-mile network buffer						
	
	Supercenter/Supermarket	Grocery Store	Convenience Store	Mass Merchandiser	Dollar Store	Pharmacy	Fast Food Restaurant
	
Need variable	b (SE)	b (SE)	b (SE)	b (SE)	b (SE)	b (SE)	b (SE)
Deprivation	-0.008 (0.017)	0.025 (0.116)	0.101 (0.080)	-0.027 (0.012)	-0.030 (0.024)	-0.001 (0.008)	-0.429 (0.161)^†^
No Vehicle	0.013 (0.005)*	0.018 (0.005)^‡^	0.012 (0.024)	-0.004 (0.004)	0.007 (0.007)	-0.004 (0.002)	0.118 (0.049)*

R^2^	0.077	0.266	0.095	0.077	0.067	0.094	0.147
*P*	0.001	<0.001	<0.001	<0.010	<0.010	<0.001	<0.001

NOTE: In this model, the seven equations were simultaneously estimated, controlling for population density. All variable entered as continuous. Results are reported as multivariate-adjusted b (SE). Statistically significant variables are indicated as: *<0.05 ^†^<0.01 ^‡^<0.001

**Table 6:** Association between 3-mile coverage and need, using multivariate linear regression

	Access as coverage with 3-mile network buffer						
	
	Supercenter/Supermarket	Grocery Store	Convenience Store	Mass Merchandiser	Dollar Store	Pharmacy	Fast Food Restaurant
	
Need variable	b (SE)	b (SE)	b (SE)	b (SE)	b (SE)	b (SE)	b (SE)
Deprivation	-0.041 (0.033)	-0.105 (0.027)^‡^	-0.078 (0.250)	-0.154 (0.022)^‡^	-0.265 (0.061)^‡^	-0.084 (0.018)^‡^	-1.830 (0.361)^‡^
No Vehicle	0.013 (0.010)	0.007 (0.008)	-0.089 (0.237)	0.003 (0.007)	-0.004 (0.018)	-0.0004 (0.005)	0.075 (0.109)

R^2^	0.041	0.167	0.038	0.318	0.200	0.229	0.241
*P*	0.046	<0.001	0.059	<0.001	<0.001	<0.001	<0.001

NOTE: In this model, the seven equations were simultaneously estimated, controlling for population density. All variable entered as continuous. Results are reported as multivariate-adjusted b (SE). Statistically significant variables are indicated as: *<0.05 ^†^<0.01 ^‡^<0.001

## Discussion

This study extends our understanding of spatial access to food resources from neighborhoods that are more socioeconomically deprived or have greater proportions of households without available transportation by examining two dimensions of access: 1) proximity (distance) to the nearest food store or fast food restaurant and 2) coverage (number) of food stores or fast food restaurants within a specified distance [40]. In contrast to studies that narrowly define the food environment as supermarkets and/or fast food restaurants [38], our examination of access from primarily *colonia *neighborhoods in an area of high or persistent poverty along the South Texas border with Mexico focuses on all food stores (i.e., supercenters, supermarkets, grocery stores, convenience stores, mass merchandisers, dollar stores, and pharmacies) and fast food restaurants.

Overall, neighborhoods had the best spatial access to a convenience store or fast food restaurant, both in the distance to the nearest convenience store or fast food restaurant and in the number of shopping opportunities at convenience stores or fast food restaurants within a certain distance of the neighborhood. Among traditional food stores, supercenter/supermarket provided greater proximity and coverage than any individual type of traditional food store. When neighborhood deprivation was considered, the results differed between proximity-determined and coverage-determined access. Using distance to the nearest store as a measure of access, CBG with increased deprivation were correlated with poor access to the nearest supercenter or grocery and good access to a convenience store. More limited availability of a vehicle was correlated with better proximity to a supercenter/supermarket, convenience store, dollar store, and fast food restaurant. However, after controlling for population density, increased deprivation was associated with greater distance to the nearest supercenter/supermarket, grocery store, mass merchandiser, dollar store, and pharmacy. Vehicle availability was not associated with any food store or fast food restaurant. This suggests that neighborhoods with greater need have poorer spatial access to the type of stores where greater selections and lower prices are available [83].

Initially, study results of coverage criterion showed that neighborhoods of greater deprivation were associated with better access to grocery stores within 1-network mile, which is opposite of the relationship using proximity. As the buffer distance increased, food store and fast food restaurant access declined with increasing deprivation. For neighborhoods with decreasing availability of a vehicle, access to food stores and fast food restaurants was greater within a 1-mile buffer, and reverse with increasing distance. After controlling for population density, neighborhoods of increased deprivation had access to fewer fast food restaurants within 1-network mile; none of the food stores was significant. At the same distance, neighborhoods with decreasing vehicle availability were more likely to have more supercenter/supermarkets, grocery stores, or fast food restaurants within one mile. At three miles, increasing deprivation was associated with fewer grocery stores, mass merchandisers, dollar stores, pharmacies, and fast food restaurants; all controlled for population density. At this same distance, vehicle availability was not associated with any aspect of the food environment.

Regardless of measure, access to the food environment, especially to supercenter/supermarkets, where the largest selections of affordable healthy foods are marketed [84], was a major problem for many residents of *colonia *neighborhoods in South Texas. Access in terms of distance to the nearest food store and the variety of stores within a given area was relatively better to convenience stores and fast food restaurants, where the opportunities for healthy foods are limited [65,85]. This study found similar pictures of spatial distribution for proximity and coverage. The value of using both measures is in the disparate information that each provides. Proximity provides a measure of network distance to the nearest store of a specific type. This can also provide a sense of travel time from starting point. Coverage describes choice; that is, the number of stores and potential difference in selection and/or price that are available within a given area.

Unlike much larger urban areas [35,40], none of the neighborhoods in this study had more than two supercenter/supermarkets within one mile and 94% did not have more than three within three miles. Being able to walk to a supermarket was out of the question for almost all the neighborhoods in this study. This compares with more urban areas in which a supermarket is walkable [38,40,42]. The difference in population density may explain much of this; for example, the Zenk study in Detroit estimated a median population density of 5367.4 while this study reports a median of 2528.9 [35].

### Strengths

There are several major strengths to this study, especially in relation to other studies. Instead of using a single measure of access, usually the distance to the nearest supermarket [35,46,86], this study used two different criteria of accessibility [40,49]. Unlike prior studies that focused on supermarkets [17,35,40,49,87], this study extends our understanding of potential spatial access of neighborhoods to a dramatically changing food environment. One example of the type of changes that continue to occur is referred to as *channel blurring*, whereby retail stores have extended their product mix into food categories not previously carried [60,88]. For example, convenience stores have extended their offerings of food items; supercenters have expanded; and growing assortments of shelf-stable and refrigerated food items have been added to dollar stores and mass merchandisers. As a result, this study presents a comprehensive look at the food environment and acknowledges the presence and contribution of different food store formats to the accessibility of food items. The exclusion of convenience stores, supercenters, dollar stores, and mass merchandisers would understate the availability of food items [65]. This is one of the few studies that used ground truthing to identify and collect on-site GPS data for all food stores and fast food restaurants within the study area. This approach provides a more complete and accurate depiction of the food environment than data from secondary sources [46]. In this study, we were able to better account for population distribution, thus minimize aggregation errors, by using population-weighted centroids, and provide more accurate access measures using network distance in calculating both minimum distance and coverage areas [46,77].

### Limitations

This study has several limitations. The distance measurement describes shopping opportunities; that is, what is potentially accessible, but does not include where people choose to shop for food items. Although public transportation is not available in all areas, available transportation routes were not included. Distance measures assume that the home is the starting point; however, individual starting points vary and may include different activities as the beginning of multiple stops that include grocery shopping [89]. Finally, the results of this study may not be generalized to other areas.

## Conclusion

Access and availability to the food environment will play a pivotal role for the nutritional health of families, especially among the increasing Hispanic population within neighborhoods of *colonias*. Many of these neighborhoods experience overall socioeconomic deprivation; many are home to large proportions of residents who have a low household income, are unemployed, or lack access to a vehicle. The lack of public transportation, especially in many of the areas, further marginalizes a large, disadvantaged population and limits their options for food resources. Indeed, it is difficult to initiate or maintain healthful eating habits without access to healthy foods. Knowing more about the food environment is essential for combining environmental approaches with traditional health interventions to make it easier for individuals to make healthier food choices [90]. The preparation for policy change to strengthen food assistance programs or program delivery activities, or interventions to improve nutritional health should include an understanding of the community – where people live and where they shop for food [90]. Additionally, it is important to understand not only the distance that people must travel to the nearest store to make a purchase, but also how much diversity in stores they have in order to compare price, quality, and selection. Future research should examine how spatial access to the food environment influences the utilization of food stores and fast food restaurants, and the strategies used by low-income Hispanic families in this area to obtain food for the household.

## Abbreviations

CBG: census block group; CFEP: Colonia Food Environment Project.

## Competing interests

The authors declare that they have no competing interests.

## Authors' contributions

JS conceptualized the study and supervised the data collection. He carried out statistical analyses. SH carried out mapping of results. JS drafted the manuscript. DH carried out spatial analysis; JCH directed multivariate modeling. All authors approved the final manuscript.

## References

[B1] White M (2007). Food access and obesity. obesity reviews.

[B2] Booth SL, Sallis JF, Ritenbaugh C, Hill JO, Birch LL, Frank LD, Glanz K, Himmelgreen DA, Mudd M, Popkin BM (2001). Environmental and Societal Factors Affect Food Choice and Physical Activity: Rationale, Influences, and Leverage Points. Nutr Rev.

[B3] Mier N, Ory MG, Zhan D, Conkling M, Sharkey JR, Burdine JN (2008). Health-related quality of life among Mexican Americans living in colonias at the Texas-Mexico border. Social Science & Medicine.

[B4] Ward PM (1999). Colonias and Public Policy in Texas and Mexico.

[B5] Bread for the World Institute (BWI) Hunger Report. http://www.bread.org/institute/hunger_report/2005-pdf.htm.

[B6] Border Health Initiative: Data Tables. http://borderhealth.cr.usgs.gov.

[B7] Texas Comptroller of Public Accounts Bordering the Future. http://www.window.state.tx.us/border/ch07/ch07.html.

[B8] McLeroy KR, Bibeau D, Steckler A, Glanz K (1988). An Ecological Perspective for Health Promotion Programs. Health Educ Q.

[B9] Robinson T (2008). Applying the Socio-ecological Model to Improving Fruit and Vegetable Intake Among Low-Income African Americans. J Community Health.

[B10] Shaw HJ (2006). Food Deserts: Towards the Development of a Classification. Geogr Ann.

[B11] Morland K, Wing S, Roux AD (2002). The Contextual Effect of the Local Food Environment on Residents' Diets: The Atherosclerosis Risk in Communities Study. Am J Public Health.

[B12] Morland K, Wing S, Roux AD, Poole C (2002). Neighborhood Characteristics Associated with the Location of Food Stores and Food Service Places. Am J Prev Med.

[B13] Furst T, Connors M, Bisogni CA, Sobal J, Falk LW (1996). Food Choice: A Conceptual Model of the Process. Appetite.

[B14] Andreyeva T, Blumenthal DM, Schwartz MB, Long MW, Brownell KD (2008). Availability and Prices of Foods Across Stores and Neighborhoods: The Case of New Haven, Connecticut. Health Affairs.

[B15] Caldwell EM, Kobayashi MM, DuBow WM, Wytinck SM (2008). Perceived access to fruits and vegetables associated with increased consumption. Public Health Nutrition.

[B16] Diez-Roux AV, Nieto FJ, Caulfied L, Tyroler HA, Watson RL, Szklo M (1999). Neighbourhood differences in diet: the Atherosclerosis Risk in Communities (ARIC) Study. J Epidemiol Community Health.

[B17] Laraia BA, Siega-Riz AM, Kaufman JS, Jones SJ (2004). Proximity of supermarkets is positively associated with diet quality index for pregnancy. Preventive Medicine.

[B18] Rose D, Richards R (2004). Food store access and household fruit and vegetable use among participants in the US Food Stamp Program. Public Health Nutrition.

[B19] Cheadle A, Psaty BM, Curry S, Wagner E, Diehr P, Koepsell T, Kristal A (1993). Can Measures of the Grocery Store Environment Be Used to Track Community-Level Dietary Changes?. Preventive Medicine.

[B20] Cheadle A, Psaty BM, Curry S, Wagner E, Diehr P, Koepsell T, Kristal A (1991). Community-Level Comparisons Between Grocery Store Environment and Individual Dietary Practices. Preventive Medicine.

[B21] Fisher B, Strogatz D (1999). Community measures of low-fat milk consumption: comparing store shelves with households. Am J Public Health.

[B22] Moore LV, Roux AVD, Nettleton JA, David R, Jacobs J (2008). Associations of the Local Food Environment with Diet Quality – A Comparison of Assessments based on Surveys and Geographic Information Systems. Am J Epidemiol.

[B23] Bodor JN, Rose D, Farley TA, Swalm C, Scott SK (2007). Neighbourhood fruit and vegetable availability and consumption: the role of small food stores in an urban environment. Public Health Nutrition.

[B24] Pearson T, Russell J, Campbell MJ, Barker ME (2005). Do 'food deserts' influence fruit and vegetable consumption? – a cross-sectional study. Appetite.

[B25] Cummins S, Petticrew M, Higgins C, Findlay A, Sparus L (2005). Large-scale food retailing as a health intervention: quasi-experimental evaluation of a natural experiment. J Epidemiol Community Health.

[B26] Wrigley N, Warm D, Margetts B (2003). Deprivation, diet and food retail access: findings from the leeds 'food deserts' study. Environ Plann A.

[B27] Morland K, Diez Roux AV, Wing S (2006). Supermarkets, Other Food Stores, and Obesity. Am J Prev Med.

[B28] Lopez R (2007). Neighborhood Risk Factors for Obesity. Obesity.

[B29] Thompson O, Ballew C, Resnicow K, Must A, Bandini L, Cyr H, Dietz W (2004). Food purchased away from home as a predictor of change in BMI *z*-score among girls. International Journal of Obesity.

[B30] Alwitt LF, Donley TD (1997). Retail Stores in Poor urban Neighborhoods. The Journal of Consumer Affairs.

[B31] Baker EA, Schootman M, Barnidge E, Kelly C (2006). The Role of Race and Poverty in Access to Foods That Enable Individuals to Adhere to Dietary Guidelines. Preventing Chronic Disease.

[B32] Block D, Kouba J (2006). A comparison of the availability and affordability of a market basket in two communities in the Chicago area. Public Health Nutrition.

[B33] Moore LV, Diez Roux AV (2006). Association of Neighborhood Characteristics With the Location and Type of Food Stores. Am J Public Health.

[B34] Powell LM, Slater S, Mirtcheva D, Bao Y, Chaloupka FJ (2007). Food store availability and neighborhood characteristics in the United States. Preventive Medicine.

[B35] Zenk SN, Schulz AJ, Israel BA, James SA, Bao S, Wilson ML (2005). Neighborhood Racial Composition, Neighborhood Poverty, and the Spatial Accessibility of Supermarkets in Metropolitan Detroit. Am J Public Health.

[B36] Horowitz CR, Colson KA, Hebert PL, Lancaster K (2004). Barriers to Buying Healthy Foods for People With Diabetes: Evidence of Environmental Disparities. Am J Public Health.

[B37] Raja S, Ma C, Yadav P (2008). Beyond Food Deserts: Measuring and Mapping Racial Disparities in Neighborhood Food Environments. Journal of Planning Education and Research.

[B38] Larsen K, Gilliland J (2008). Mapping the evolution of 'food deserts' in a Canadian city: Supermarket accessibility in London, Ontario, 1961–2005. International Journal of Health Geographics.

[B39] Ball K, Timperio A, Crawford D (2009). Neighbourhood socioeconomic inequalities in food access and availability. Health & Place.

[B40] Apparicio P, Cloutier M-S, Shearmur R (2007). The case of Montreal's missing food deserts: Evaluation of accessibility to food supermarkets. International Journal of Health Geographics.

[B41] Latham J, Moffat T (2007). Determinants of variation in food cost and availability in two socioeconomically contrasting neighborhoods of Hamilton, Ontario, Canada. Health & Place.

[B42] Smoyer-Tomic KE, Spence JC, Raine KD, Amrhein C, Cameron N, Yasenovskiy V, Cutumisu N, Hemphill E, Healy J (2008). The association between neighborhood socioeconomic status and exposure to supermarkets and fast food outlets. Health & Place.

[B43] Travers K, Cogdon A, McDonald W, Wright C, Anderson B, MacLean D (1997). Availability and cost of heart healthy dietary changes in Nova Scotia. J Can Diet Assoc.

[B44] Winkler E, Turrell G, Patterson C (2006). Does living in a disadvantaged area mean fewer opportunities to purchase fresh fruit and vegetables in the area? Findings from the Brisbane food study. Health & Place.

[B45] Pearce J, Hiscock R, Blakely T, Witten K (2008). The contextual effects of neighbourhood access to supermarkets and convenience stores on individual fruit and vegetable consumption. J Epidemiol Community Health.

[B46] Sharkey J, Horel S (2008). Neighborhood Socioeconomic Deprivation and Minority Composition Are Associated with Better Potential Spatial Access to the Food Environment in a Large Rural Area. J Nutr.

[B47] Cassady D, Jetter KM, Culp J (2007). Is Price a Barrier to Eating More Fruits and Vegetables for Low-Income Families?. J Am Diet Assoc.

[B48] Pearce J, Day P, Witten K (2008). Neighbourhood Provision of Food and Alcohol Retailing and Social Deprivation in Urban New Zealand. Urban Policy and Research.

[B49] Smoyer-Tomic KE, Spence JC, Amrhein C (2006). Food Deserts in the Prairies? supermarket Accessibility and Neighborhood Need in Edmonton, Canada. The Professional Geographer.

[B50] Inagami S, Cohen DA, Finch BK, Asch SM (2006). You Are Where You Shop: Grocery Store Locations, Weight, and Neighborhoods. Am J Prev Med.

[B51] Kaufman PR, Lyson TA Retail Concentration, Food Deserts, and Food Disadvantaged Communities in Rural America. http://srdc.msstate.edu/focusareas/health/fa/blanchard02_final.pdf.

[B52] Kaufman PR, MacDonald JM, Lutz SM, Smallwood DM (1997). Do the Poor Pay More for Food? Item Selection and Price Differences Affect Low-Income Household Food Costs.

[B53] Yadrick K, Horton J, Stuff J, McGee B, Bogle M, Davis L, Forrester I, Strickland E, Casey PH, Ryan D (2001). Perceptions of Community Nutrition and Health Needs in the Lower Mississippi Delta: A Key Informant Approach. JNE.

[B54] Liese AD, Weis KE, Pluto D (2007). Food store types, availability and cost of foods in a rural environment. J Am Diet Assoc.

[B55] Cummins SC, McKay L, MacIntyre S (2005). McDonald's Restaurants and Neighborhood Deprivation in Scotland and England. Am J Prev Med.

[B56] Donkin AJ, Dowler EA, Stevenson SJ, Turner SA (2000). Mapping access to food in a deprived area: the development of price and availability indices. Public Health Nutrition.

[B57] Macdonald L, Cummins S, Macintyre S (2007). Neighbourhood fast food environment and area deprivation – substitution or concentration?. Appetite.

[B58] Messer LC, Laraia BA, Kaufman JS, Eyster J, Holzman C, Culhane J, Elo I, Burke JG, O'Campo P (2006). The Development of a Standardized Neighborhood Deprivation Index. Journal of Urban Health: Bulletin of the New York Academy of Medicine.

[B59] Pearce J, Blakely T, Witten K, Bartie P (2007). Neighborhood Deprivation and Access to Fast-Food Retailing: A National Study. Am J Prev Med.

[B60] Leibtag ES (2005). Where You Shop Matters: Store Formats Drive Variation in Retail Food Prices. AmberWaves.

[B61] Fast Food and Quickservice Restaurants. http://www.hoovers.com/fast-food-&-quick-service-restaurants/--HICID_1444--/free-ind-factsheet.xhtml.

[B62] Powell LM, Chaloupka FJ, Bao Y (2007). The Availability of Fast-Food and Full-Service Restaurants in the United States. Am J Prev Med.

[B63] U.S. Census Bureau State and County QuickFacts. http://quickfacts.census.gov/qfd/states/48/48215.html.

[B64] U.S. Census Bureau North American Industry Classification System (NAICS). http://www.census.gov/epcd/www/naics.html.

[B65] Hale T (2004). Dollar Store, No Frills: The New Retail Landscape. Consumer Insight.

[B66] Sharkey JR, Sharf BF, John JS Participant-Observationsto Focus on Eating Behaviors and Patterns among *Colonia *Families. 2007 Annual Meeting of the International Society for Behavioral Nutrition and Physical Activity, June 20–23, 2007.

[B67] French S, Story M, Neumark-Sztainer D, Fulkerson J, Hannan P (2001). Fast food restaurant use among adolescents: associations with nutrient intake, food choices and behavioral and psychosocial variables. International Journal of Obesity.

[B68] Jeffery RW, Baxter J, McGuire M, Linde J (2006). Are fast food restaurants an environmental risk factor for obesity?. International Journal of Behavioral Nutrition and Physical Activity.

[B69] Kyureghian G, Rodolfo M, Nayga J, Davis GC, Lin B-H (2007). Obesity and Food Away from Home Consumption by Restaurant Type and Meal Occasion: Can Fast Food and Lunch Away from Home Male You Fatter?.

[B70] Witte TH, Wilson AM (2005). Accuracy of WAAS-enabled GPS for determination of position and speed over ground. Journal of Biomechanics.

[B71] Garmin Ltd About GPS. http://www8.garmin.com/aboutGPS/waas.html.

[B72] Winkleby M, Cubbin C, Ahn D (2006). Effect of Cross-Level Interaction Between Individual and Neighborhood Socioeconomic Status on Adult Mortality Rates. Am J Public Health.

[B73] U.S. Census Bureau Geographic Areas Reference Manual. http://www.census.gov/geo/www/garm.html.

[B74] Hanigan I, Hall G, Dear KB (2006). A comparison of methods for calculating population exposure estimates of daily weather for health research. International Journal of Health Geographics.

[B75] Martinez SW (2007). The U.S. Food Marketing System: Recent Developments, 1997–2006. Economic Research Report Number 42, USDA Economic Research Service.

[B76] Smoyer-Tomic KE, Hewko JN, Hodgson MJ (2004). Spatial accessibility and equity of playgrounds in Edmonton, Canada. The Canadian Geographer.

[B77] Sampson RJ, Raudenbush SW, Earls F (1997). Neighborhoods and Violent Crime: A Multilevel Study of Collective Efficacy. Science.

[B78] Matheson FI, Moineddin R, Dunn JR, Creatore MI, Gozdyra P, Glazier RH (2006). Urban neighborhoods, chronic stress, gender and depression. Soc Sci Med.

[B79] Wen M, Hawkley LC, Cacioppo JT (2006). Objective and perceived neighborhood environment, individual SES and psychosocial factors, and self-rated health: An analysis of older adults in Cook County, Illinois. Soc Sci Med.

[B80] Kim J, Mueller CW (1978). Factor analysis: Statistical methods and practical issues.

[B81] Anselin L, Syabri I, Kho Y (2006). GeoDa: An Introduction to Spatial Data Analysis. Geographical Analysis.

[B82] Morland K, Filomena S (2007). Disparities in the availability of fruits and vegetables between racially segregated urban neighborhoods. Public Health Nutrition.

[B83] Creel JS, Sharkey JR, McIntosh A, Anding J, Huber JC (2008). Availability of Healthier Options in Traditional and Nontraditional Rural Fast-Food Outlets. BMC Public Health.

[B84] Bustillos BD, Sharkey JR, Anding J, McIntosh A (2009). Availability of healthier food alternatives in traditional, convenience, and non-traditional types of food stores in two rural Texas counties. J Am Diet Assoc.

[B85] Jago R, Baranowski T, Baranowski JC, Cullen KW, Thompson D (2007). Distance to food stores & adolescent male fruit and vegetable consumption: mediation effects. International Journal of Behavioral Nutrition and Physical Activity.

[B86] Kaufman PR (1998). Rural Poor Have Less Access to Supermarkets, Large grocery Stores. Rural Development Perspectives.

[B87] Shih W, Kaufman S, McKillican R Dollar General (A).

[B88] McGuckin N, Nakamoto R Trips, Chains, and Tours – Using an Operational Definition. http://www.trb.org/conferences/nhts/McGuckin.pdf.

[B89] Seymour JD, Yaroch AL, Serdula M, Blanck HM, Khan LK (2004). Impact of nutrition environmental interventions on point-of-purchase behavior in adults: a review. Preventive Medicine.

[B90] Gesler WM, Hayes M, Arcury TA, Skelly AH, Nash S, Soward AC (2004). Use of mapping technology in health intervention research. Nurs Outlook.

